# 2-(10′,10′-Dimethyl-3′-sulfanyl­idene-4′-aza­tri­cyclo­[5.2.1.0^1,5^]decan-2′­-yl)-10,10-dimethyl-4-aza­tri­cyclo­[5.2.1.0^1,5^]decane-3-thione

**DOI:** 10.1107/S1600536813019211

**Published:** 2013-07-20

**Authors:** Ashley Walker, Craig M. Forsyth, Patrick Perlmutter

**Affiliations:** a School of Chemistry, Monash University, Clayton, Victoria 3800, Australia

## Abstract

The title compound, C_28_H_40_N_2_O_2_S_2_, was obtained as a minor product from an *anti*-aldol reaction between the corresponding *N*-propionyl­thiol­actam and benzaldehyde. The asymmetric unit contains one half-molecule, which is completed by inversion symmetry. The molecule displays a nearly eclipsed conformation along the central C—C bond with a C—C—C—C— torsion angle of 20.4 (3)°.

## Related literature

For chiral auxiliaries providing control over the sterochemical outcome of chemical transformations, see: Valezquez & Olivo (2002 ▶). For a related synthesis, see: Tamaru *et al.* (1978 ▶).
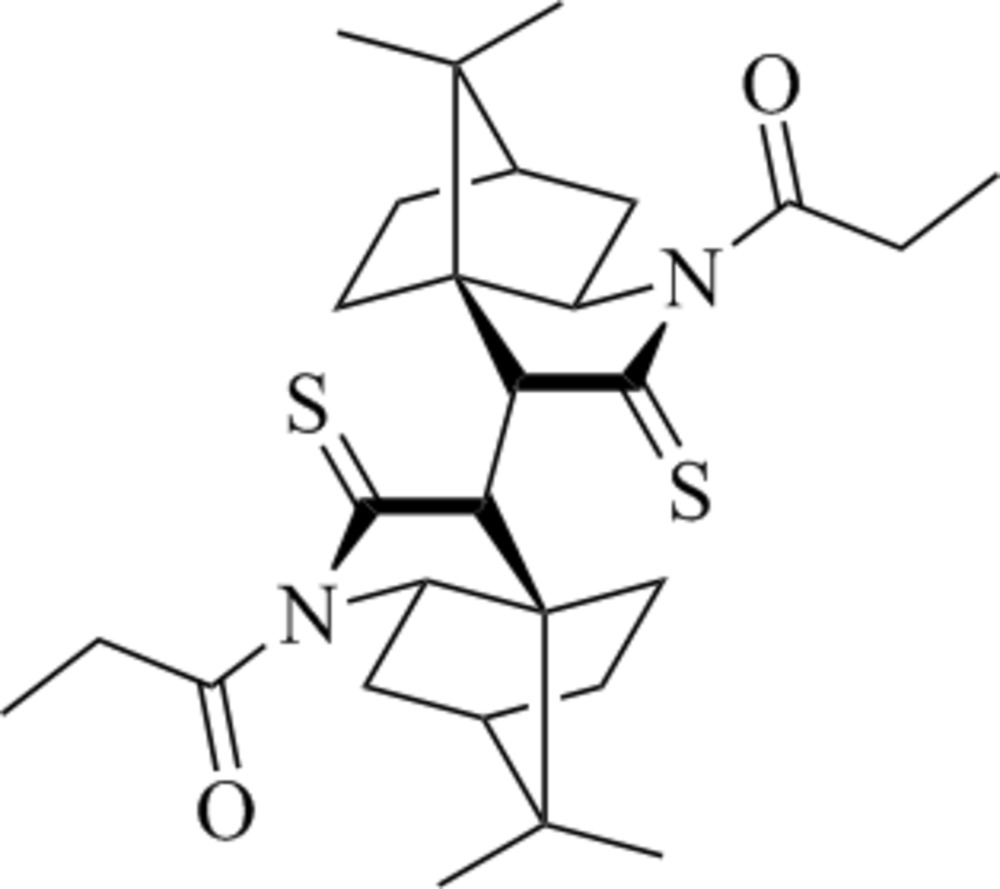


## Experimental

### 

#### Crystal data

C_28_H_40_N_2_O_2_S_2_
*M* *_r_* = 500.74
Tetragonal, 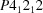

*a* = 13.8159 (3) Å
*c* = 13.5221 (6) Å
*V* = 2581.09 (16) Å^3^
*Z* = 4
Mo *K*α radiation
μ = 0.24 mm^−1^
*T* = 123 K
0.20 × 0.15 × 0.08 mm

#### Data collection

Bruker X8 APEX CCD diffractometer
Absorption correction: multi-scan (*SADABS*; Bruker, 2004 ▶) *T*_min_ = 0.94, *T*_max_ = 0.98
18157 measured reflections
3814 independent reflections
3601 reflections with *I* > 2σ(*I*)
*R*_int_ = 0.052

#### Refinement

*R*[*F* ^2^ > 2σ(*F* ^2^)] = 0.049
*wR*(*F* ^2^) = 0.100
*S* = 1.19
3814 reflections
154 parameters
H-atom parameters constrained
Δρ_max_ = 0.26 e Å^−3^
Δρ_min_ = −0.30 e Å^−3^
Absolute structure: Parsons & Flack (2004 ▶); Flack x determined using 1367 quotients [(I+)-(I-)]/[(I+)+(I-)]
Absolute structure parameter: 0.04 (4)

### 

Data collection: *APEX2* (Bruker, 2006 ▶); cell refinement: *SAINT* (Bruker, 2006 ▶); data reduction: *SAINT*; program(s) used to solve structure: *SHELXS97* (Sheldrick, 2008 ▶); program(s) used to refine structure: *SHELXL2013* (Sheldrick, 2008 ▶); molecular graphics: *X-SEED* (Barbour, 2001 ▶); software used to prepare material for publication: *CIFTAB* (Sheldrick, 1997 ▶).

## Supplementary Material

Crystal structure: contains datablock(s) global, I. DOI: 10.1107/S1600536813019211/gw2136sup1.cif

Structure factors: contains datablock(s) I. DOI: 10.1107/S1600536813019211/gw2136Isup2.hkl

Additional supplementary materials:  crystallographic information; 3D view; checkCIF report

## Figures and Tables

**Fig. 1.**
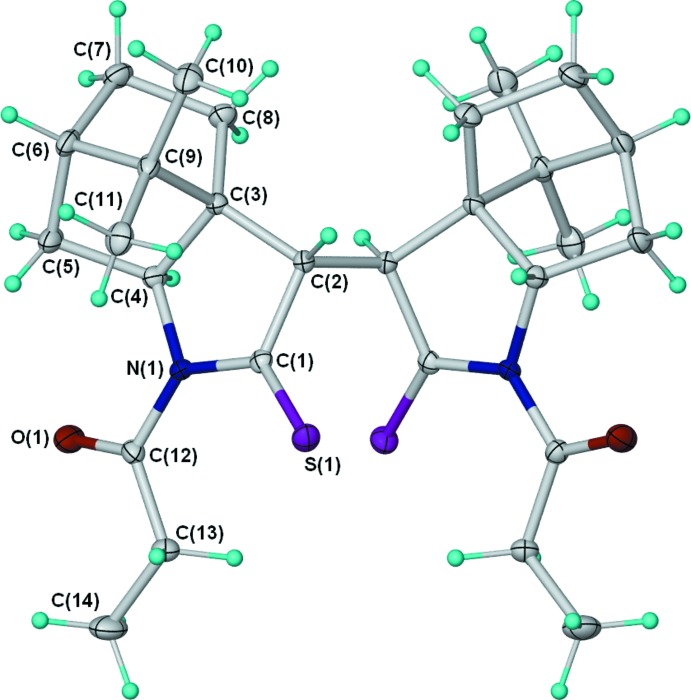
Molecular diagram of the title compound. Displacement ellipsoids are drawn at the 50% probability level.

## supplementary crystallographic information

### Comment

Chiral auxiliaries provide control over the sterochemical outcome of chemical transformations (Valezquez & Olivo, 2002). During the course of studies on a new type of chiral auxilliary, dimer I was obtained as the unexpected product of a Et_2_BOTf-promoted *anti*-aldol reaction between the corresponding *N*-propionyl thiolactam and benzaldehyde. We postulate that the dimeristion occurs *via* initial deprotonation of the thiolactam α-carbon to give a thioenolate, followed by oxidative coupling and eventual [3,3]sigmatropic rearrangement of the resultant disulfide (Tamaru *et al.*, 1978). The molecular structure comprises one half of the dimer in the ASU, the other half generated by the symmetry operator #1 - *y*,-*x*,1/2 - *z*. The thiolactam ring is non-planar with an S configuration at C(2) and a nearly eclipsed conformation along the central C—C bond (C(1)—C(2)—C(2)#1-C(1)#1 20.4 (3)°).

### Experimental

A freshly prepared solution of diethylboron trifluoromethanesulfonate in hexane (from triflic acid (60µ*L*, 0.67 mmol) and triethylborane (0.67µ*L* of a 1*M* solution in hexane, 0.67 mmol)) was cooled to -5 °C. A solution of *N*-propionyl-10',10'-dimethyl-4'-azatricyclo[5.2.1.0^1,5^]decan -3'-thione (85 mg, 0.34 mmol) in CH_2_Cl_2_ (1.5 mL) was added dropwise, whilst maintaining temperature below 0 °C. After 10 min, *i*-Pr_2_NEt (150µ*L*, 0.84 mmol) was added dropwise and the reaction mixture was stirred for 30 min. After cooling to -78 °C, benzaldehyde (110/ml, 1.01 mmol) was added dropwise. The reaction was quenched by dropwise addition of pH 6 phosphate buffer (5 mL) and the resulting mixture was allowed to warm to room tempersture. The organic fraction was extracted into CH_2_Cl_2_ (3x5mL)and the combined extracts were dried over MgSO_4_, filtered and the solvent removed *in vacuo*. The yellow residue was purified by flash chromatography (3:2 hexane/Et_2_O) to afford the *title compound* as yellow prisms. ^1^H NMR (300 MHz, CDCl_3_δ, p.p.m.): 0.91 (s, 6H), 0.93 (s, 6H), 1.11 (t, *J* 7.2 Hz, 6H), 1.41 (m, 2H), 1.71 (m, 2H), 2.07 (m, 4H), 3.04 (q, *J* 7.2 Hz, 4H), 3.19 (s, 2H), 4.62 (dd, *J* 5.4, 7.9 Hz, 2H). ^13^C NMR (75 MHz, CDCl_3_δ, p.p.m.): 8.58, 19.82, 20.63, 26.82, 29.07, 33.75, 38.27, 45.38, 48.62, 55.45, 61.33, 74.22, 175.65, 211.55. IR (ν, cm^-1^): 2960(*s*), 1695(*s*), 1458(w), 1405(w), 1377(*m*), 1352(*m*), 1305(*m*), 1275(*m*), 1217(*m*), 1152(*m*), 1110(*m*), 1075(*m*), 1048(*m*), 958(w), 808(w), 707(w), 626(w). HRMS: Calcd for (C_28_H_41_N_2_O_2_S_2_)^+^*m/z* 501.2609; Found 501.2661

### Refinement

All H atoms for the primary molecules were initially located in the difference Fourier map but were placed in geometrically idealized positions and constrained to ride on their parent atoms with C—H distances in the range 0.95–1.00 Å and *U*_iso_(H) = 1.2–1.5 *U*_eq_(C).

### Crystal data

**Table:**

C_28_H_40_N_2_O_2_S_2_	*D*_x_ = 1.289 Mg m^−^^3^
*M**_r_* = 500.74	Mo *K*α radiation, λ = 0.71073 Å
Tetragonal, *P*4_1_2_1_2	Cell parameters from 4395 reflections
*a* = 13.8159 (3) Å	θ = 2.6–29.9°
*c* = 13.5221 (6) Å	µ = 0.24 mm^−^^1^
*V* = 2581.09 (16) Å^3^	*T* = 123 K
*Z* = 4	Prism, colourless
*F*(000) = 1080	0.20 × 0.15 × 0.08 mm

### Data collection

**Table:**

Bruker X8 APEX CCD diffractometer	3814 independent reflections
Radiation source: fine-focus sealed tube	3601 reflections with *I* > 2σ(*I*)
Graphite monochromator	*R*_int_ = 0.052
thin slice φ and ω scans	θ_max_ = 30.1°, θ_min_ = 2.1°
Absorption correction: multi-scan (*SADABS*; Bruker, 2004)	*h* = −19→15
*T*_min_ = 0.94, *T*_max_ = 0.98	*k* = −18→19
18157 measured reflections	*l* = −19→18

### Refinement

**Table:**

Refinement on *F*^2^	Hydrogen site location: inferred from neighbouring sites
Least-squares matrix: full	H-atom parameters constrained
*R*[*F*^2^ > 2σ(*F*^2^)] = 0.049	*w* = 1/[σ^2^(*F*_o_^2^) + (0.0302*P*)^2^ + 0.8743*P*] where *P* = (*F*_o_^2^ + 2*F*_c_^2^)/3
*wR*(*F*^2^) = 0.100	(Δ/σ)_max_ < 0.001
*S* = 1.19	Δρ_max_ = 0.26 e Å^−^^3^
3814 reflections	Δρ_min_ = −0.30 e Å^−^^3^
154 parameters	Absolute structure: Parsons & Flack (2004); Flack x determined using 1367 quotients [(I+)-(I-)]/[(I+)+(I-)]
0 restraints	Flack parameter: 0.04 (4)

### Special details

**Table:**

Geometry. All e.s.d.'s (except the e.s.d. in the dihedral angle between two l.s. planes) are estimated using the full covariance matrix. The cell e.s.d.'s are taken into account individually in the estimation of e.s.d.'s in distances, angles and torsion angles; correlations between e.s.d.'s in cell parameters are only used when they are defined by crystal symmetry. An approximate (isotropic) treatment of cell e.s.d.'s is used for estimating e.s.d.'s involving l.s. planes.

### Fractional atomic coordinates and isotropic or equivalent isotropic displacement parameters (Å^2^)

**Table:**

	*x*	*y*	*z*	*U*_iso_*/*U*_eq_	
S1	−0.06533 (5)	0.15852 (5)	0.37365 (5)	0.01864 (15)	
O1	0.06705 (15)	0.29052 (14)	0.09532 (15)	0.0238 (4)	
N1	0.05153 (14)	0.17685 (14)	0.21226 (16)	0.0132 (4)	
C1	0.00988 (17)	0.12335 (18)	0.28611 (19)	0.0129 (5)	
C2	0.04889 (17)	0.02031 (17)	0.27992 (19)	0.0127 (5)	
H2	0.0589	−0.0060	0.3481	0.015*	
C3	0.14660 (17)	0.03484 (17)	0.22943 (18)	0.0128 (5)	
C4	0.12967 (17)	0.12247 (18)	0.16116 (19)	0.0145 (5)	
H4	0.1074	0.1005	0.0945	0.017*	
C5	0.23097 (18)	0.1709 (2)	0.1537 (2)	0.0199 (6)	
H5A	0.2513	0.1787	0.0840	0.024*	
H5B	0.2318	0.2348	0.1868	0.024*	
C6	0.29552 (18)	0.09742 (19)	0.2084 (2)	0.0188 (5)	
H6	0.3607	0.1231	0.2270	0.023*	
C7	0.2986 (2)	0.0036 (2)	0.1471 (2)	0.0218 (6)	
H7A	0.3502	−0.0403	0.1712	0.026*	
H7B	0.3098	0.0177	0.0763	0.026*	
C8	0.19577 (19)	−0.04196 (19)	0.1637 (2)	0.0186 (5)	
H8A	0.1608	−0.0504	0.1004	0.022*	
H8B	0.2001	−0.1052	0.1979	0.022*	
C9	0.23189 (17)	0.0686 (2)	0.29719 (19)	0.0163 (5)	
C10	0.2748 (2)	−0.0132 (2)	0.3598 (2)	0.0230 (6)	
H10A	0.2305	−0.0285	0.4142	0.035*	
H10B	0.3374	0.0074	0.3867	0.035*	
H10C	0.2839	−0.0707	0.3185	0.035*	
C11	0.21142 (19)	0.1521 (2)	0.3678 (2)	0.0224 (6)	
H11A	0.1701	0.1294	0.4219	0.034*	
H11B	0.1785	0.2043	0.3320	0.034*	
H11C	0.2726	0.1764	0.3949	0.034*	
C12	0.02120 (18)	0.26367 (18)	0.1666 (2)	0.0159 (5)	
C13	−0.06707 (19)	0.31563 (18)	0.2041 (2)	0.0184 (5)	
H13A	−0.0560	0.3367	0.2731	0.022*	
H13B	−0.1230	0.2708	0.2037	0.022*	
C14	−0.0900 (2)	0.4034 (2)	0.1403 (2)	0.0304 (7)	
H14A	−0.1476	0.4361	0.1664	0.046*	
H14B	−0.1023	0.3825	0.0722	0.046*	
H14C	−0.0350	0.4481	0.1413	0.046*	

### Atomic displacement parameters (Å^2^)

**Table:**

	*U*^11^	*U*^22^	*U*^33^	*U*^12^	*U*^13^	*U*^23^
S1	0.0187 (3)	0.0188 (3)	0.0184 (3)	0.0002 (2)	0.0045 (3)	−0.0031 (3)
O1	0.0213 (10)	0.0246 (10)	0.0256 (10)	0.0043 (8)	0.0038 (9)	0.0106 (8)
N1	0.0103 (9)	0.0134 (9)	0.0158 (10)	0.0010 (7)	0.0003 (8)	0.0006 (8)
C1	0.0112 (11)	0.0143 (11)	0.0132 (11)	−0.0012 (8)	−0.0024 (9)	−0.0006 (9)
C2	0.0121 (11)	0.0119 (11)	0.0140 (11)	−0.0005 (8)	0.0005 (9)	0.0010 (9)
C3	0.0113 (11)	0.0128 (10)	0.0144 (12)	0.0004 (8)	0.0001 (9)	0.0020 (9)
C4	0.0145 (11)	0.0152 (11)	0.0140 (11)	0.0020 (9)	0.0019 (9)	0.0026 (9)
C5	0.0139 (11)	0.0214 (13)	0.0245 (14)	0.0010 (10)	0.0030 (10)	0.0088 (11)
C6	0.0117 (11)	0.0227 (13)	0.0219 (14)	−0.0007 (9)	0.0026 (10)	0.0064 (11)
C7	0.0161 (12)	0.0245 (14)	0.0249 (15)	0.0044 (10)	0.0058 (11)	0.0037 (12)
C8	0.0174 (12)	0.0186 (13)	0.0197 (13)	0.0028 (10)	0.0048 (10)	−0.0011 (10)
C9	0.0117 (10)	0.0183 (12)	0.0188 (12)	−0.0019 (10)	−0.0023 (10)	0.0051 (11)
C10	0.0192 (13)	0.0268 (14)	0.0232 (16)	−0.0003 (10)	−0.0031 (11)	0.0068 (11)
C11	0.0192 (12)	0.0255 (13)	0.0224 (14)	−0.0048 (10)	−0.0042 (11)	−0.0016 (12)
C12	0.0146 (11)	0.0136 (11)	0.0195 (13)	−0.0007 (9)	−0.0021 (10)	0.0029 (10)
C13	0.0158 (12)	0.0167 (12)	0.0227 (13)	0.0031 (10)	−0.0008 (11)	0.0017 (10)
C14	0.0264 (15)	0.0270 (15)	0.0378 (18)	0.0123 (12)	0.0026 (13)	0.0085 (13)

### Geometric parameters (Å, º)

**Table:**

S1—C1	1.648 (3)	C7—C8	1.570 (4)
O1—C12	1.211 (3)	C7—H7A	0.9900
N1—C1	1.369 (3)	C7—H7B	0.9900
N1—C12	1.413 (3)	C8—H8A	0.9900
N1—C4	1.486 (3)	C8—H8B	0.9900
C1—C2	1.524 (3)	C9—C11	1.524 (4)
C2—C3	1.526 (3)	C9—C10	1.531 (4)
C2—C2^i^	1.576 (5)	C10—H10A	0.9800
C2—H2	1.0000	C10—H10B	0.9800
C3—C4	1.540 (3)	C10—H10C	0.9800
C3—C8	1.542 (3)	C11—H11A	0.9800
C3—C9	1.564 (3)	C11—H11B	0.9800
C4—C5	1.554 (3)	C11—H11C	0.9800
C4—H4	1.0000	C12—C13	1.503 (4)
C5—C6	1.540 (4)	C13—C14	1.521 (4)
C5—H5A	0.9900	C13—H13A	0.9900
C5—H5B	0.9900	C13—H13B	0.9900
C6—C7	1.539 (4)	C14—H14A	0.9800
C6—C9	1.540 (4)	C14—H14B	0.9800
C6—H6	1.0000	C14—H14C	0.9800
			
C1—N1—C12	130.7 (2)	H7A—C7—H7B	109.0
C1—N1—C4	111.81 (19)	C3—C8—C7	101.8 (2)
C12—N1—C4	116.2 (2)	C3—C8—H8A	111.4
N1—C1—C2	108.4 (2)	C7—C8—H8A	111.4
N1—C1—S1	129.01 (19)	C3—C8—H8B	111.4
C2—C1—S1	122.52 (19)	C7—C8—H8B	111.4
C1—C2—C3	102.38 (18)	H8A—C8—H8B	109.3
C1—C2—C2^i^	112.35 (13)	C11—C9—C10	106.5 (2)
C3—C2—C2^i^	112.8 (2)	C11—C9—C6	113.5 (2)
C1—C2—H2	109.7	C10—C9—C6	113.6 (2)
C3—C2—H2	109.7	C11—C9—C3	116.9 (2)
C2^i^—C2—H2	109.7	C10—C9—C3	113.3 (2)
C2—C3—C4	103.72 (19)	C6—C9—C3	92.89 (19)
C2—C3—C8	123.9 (2)	C9—C10—H10A	109.5
C4—C3—C8	105.2 (2)	C9—C10—H10B	109.5
C2—C3—C9	116.3 (2)	H10A—C10—H10B	109.5
C4—C3—C9	103.4 (2)	C9—C10—H10C	109.5
C8—C3—C9	102.2 (2)	H10A—C10—H10C	109.5
N1—C4—C3	103.25 (19)	H10B—C10—H10C	109.5
N1—C4—C5	117.8 (2)	C9—C11—H11A	109.5
C3—C4—C5	103.89 (19)	C9—C11—H11B	109.5
N1—C4—H4	110.4	H11A—C11—H11B	109.5
C3—C4—H4	110.4	C9—C11—H11C	109.5
C5—C4—H4	110.4	H11A—C11—H11C	109.5
C6—C5—C4	102.0 (2)	H11B—C11—H11C	109.5
C6—C5—H5A	111.4	O1—C12—N1	116.9 (2)
C4—C5—H5A	111.4	O1—C12—C13	123.1 (2)
C6—C5—H5B	111.4	N1—C12—C13	119.9 (2)
C4—C5—H5B	111.4	C12—C13—C14	111.0 (2)
H5A—C5—H5B	109.2	C12—C13—H13A	109.4
C7—C6—C5	108.2 (2)	C14—C13—H13A	109.4
C7—C6—C9	102.6 (2)	C12—C13—H13B	109.4
C5—C6—C9	102.3 (2)	C14—C13—H13B	109.4
C7—C6—H6	114.1	H13A—C13—H13B	108.0
C5—C6—H6	114.1	C13—C14—H14A	109.5
C9—C6—H6	114.1	C13—C14—H14B	109.5
C6—C7—C8	103.6 (2)	H14A—C14—H14B	109.5
C6—C7—H7A	111.0	C13—C14—H14C	109.5
C8—C7—H7A	111.0	H14A—C14—H14C	109.5
C6—C7—H7B	111.0	H14B—C14—H14C	109.5
C8—C7—H7B	111.0		
			
C12—N1—C1—C2	158.2 (2)	C4—C5—C6—C9	−41.2 (3)
C4—N1—C1—C2	−8.3 (3)	C5—C6—C7—C8	−73.3 (3)
C12—N1—C1—S1	−24.9 (4)	C9—C6—C7—C8	34.4 (3)
C4—N1—C1—S1	168.63 (19)	C2—C3—C8—C7	−171.0 (2)
N1—C1—C2—C3	25.5 (2)	C4—C3—C8—C7	70.4 (2)
S1—C1—C2—C3	−151.66 (18)	C9—C3—C8—C7	−37.3 (2)
N1—C1—C2—C2^i^	−95.8 (3)	C6—C7—C8—C3	2.1 (3)
S1—C1—C2—C2^i^	87.1 (3)	C7—C6—C9—C11	−176.3 (2)
C1—C2—C2^i^—C1^i^	−20.4 (3)	C5—C6—C9—C11	−64.1 (3)
C1—C2—C3—C4	−31.9 (2)	C7—C6—C9—C10	61.8 (3)
C2^i^—C2—C3—C4	89.1 (2)	C5—C6—C9—C10	174.0 (2)
C1—C2—C3—C8	−151.2 (2)	C7—C6—C9—C3	−55.2 (2)
C2^i^—C2—C3—C8	−30.2 (3)	C5—C6—C9—C3	57.0 (2)
C1—C2—C3—C9	80.8 (2)	C2—C3—C9—C11	−46.9 (3)
C2^i^—C2—C3—C9	−158.2 (2)	C4—C3—C9—C11	66.0 (3)
C1—N1—C4—C3	−12.4 (3)	C8—C3—C9—C11	175.1 (2)
C12—N1—C4—C3	179.0 (2)	C2—C3—C9—C10	77.6 (3)
C1—N1—C4—C5	−126.1 (2)	C4—C3—C9—C10	−169.5 (2)
C12—N1—C4—C5	65.2 (3)	C8—C3—C9—C10	−60.4 (3)
C2—C3—C4—N1	27.5 (2)	C2—C3—C9—C6	−165.2 (2)
C8—C3—C4—N1	158.87 (19)	C4—C3—C9—C6	−52.2 (2)
C9—C3—C4—N1	−94.3 (2)	C8—C3—C9—C6	56.9 (2)
C2—C3—C4—C5	151.0 (2)	C1—N1—C12—O1	−172.5 (2)
C8—C3—C4—C5	−77.6 (2)	C4—N1—C12—O1	−6.5 (3)
C9—C3—C4—C5	29.2 (2)	C1—N1—C12—C13	4.8 (4)
N1—C4—C5—C6	120.1 (2)	C4—N1—C12—C13	170.8 (2)
C3—C4—C5—C6	6.7 (3)	O1—C12—C13—C14	0.7 (4)
C4—C5—C6—C7	66.7 (3)	N1—C12—C13—C14	−176.4 (2)

Symmetry code: (i) −*y*, −*x*, −*z*+1/2.
